# In Vitro and In Vivo Antitumor Activity of *Lophocereus marginatus* (DC.) S. Arias & Terrazas Endophytic *Aspergillus versicolor* and *Metarhizium anisopliae* Extracts Against the Murine Lymphoma L5178Y-R

**DOI:** 10.3390/microorganisms12112310

**Published:** 2024-11-13

**Authors:** Diana Laura Clark-Pérez, César Iván Romo-Sáenz, Jesica María Ramírez-Villalobos, Patricia Tamez-Guerra, Diana Caballero-Hernández, Ana Laura Delgado-Miranda, Andrés García, Joel Horacio Elizondo-Luevano, Cristina Rodríguez-Padilla, Ricardo Gomez-Flores

**Affiliations:** 1 Universidad Autónoma de Nuevo León, Facultad de Ciencias Biológicas, Departamento de Microbiología e Inmunología, San Nicolás de los Garza 66455, Nuevo León, Mexico; diana.clarkp@uanl.edu.mx (D.L.C.-P.); jesicamrv_901@hotmail.com (J.M.R.-V.); patricia.tamezgr@uanl.edu.mx (P.T.-G.); diana.caballerohr@uanl.edu.mx (D.C.-H.); laura.delgadom@uanl.edu.mx (A.L.D.-M.); joel.elizondolv@uanl.edu.mx (J.H.E.-L.); crrodrig07@gmail.com (C.R.-P.); 2 Universidad Autónoma del Estado de Morelos Centro de Investigación en Biotecnología, Laboratorio de Biotecnología Ambiental, Av. Universidad 1001, Col. Chamilpa, Cuernavaca 62209, Morelos, Mexico; andres.garcia@uaem.edu.mx

**Keywords:** anticancer, medicinal plants, extracts, lymphoma, mice survival

## Abstract

Cancer belongs to a group of diseases characterized by uncontrolled cell growth. The search for new effective treatments for cancer has led to the discovery of different molecules from plants, bacteria, and fungi with pharmacological use. Plant endophytic fungi are large producers of metabolites with antitumor properties. We aimed to evaluate the in vitro and in vivo antitumor potential of extracts from *Lophocereus marginatus* endophytic fungi. We obtained ethyl acetate and hexane extracts from the *L. marginatus* endophytes *Metarhizium anisopliae* and *Aspergillus versicolor* and evaluated their antitumor activity against murine L5178Y-R lymphoma cells and human peripheral blood mononuclear cells, using the 3-(4,5-dimethylthiazol-2-yl)-2-diphenyltetrazolium bromide reduction colorimetric technique. *M. anisopliae* and *A. versicolor* ethyl acetate extracts showed IC_50_ values of 9.168 ± 1.21 μg/mL and 13.51 ± 1.62, respectively, and selectivity indices > 30. We also observed that the maximum tolerated dose (100 mg/kg) of ethyl acetate extracts and the vehicle in BALB/c mice did not cause hepatotoxicity. In addition, we evaluated the effects of ethyl acetate extracts on survival and tumor volume in the L5178Y-R lymphoma tumor model. An increase in survival (17 d) was observed in mice treated with *A. versicolor* extract. Furthermore, it did not increase tumor volume during 10 d, as compared with the control groups without treatment, vehicle, and *M. anisopliae* extract, which had a maximum survival of 10 d. *A. versicolor* ethyl acetate extract showed in vitro and in vivo antitumor activity against lymphoma L5178Y-R, increasing mice survival.

## 1. Introduction

Cancer is a disease where cells lose their normal characteristics and functions, presenting proximally or distally uncontrolled growth, which may originate from inherited or acquired mutations [1]. In particular, lymphoma is made up of different cancers of the lymphatic system and derives from immune cells throughout their stages of differentiation. It represents one of the cancers with the highest incidence worldwide [2]. The most common and aggressive type of lymphoma is non-Hodgkin lymphoma, which is characterized by disorders in the proliferation of T, B, and NK cells [3].

Because of the complexity and variability of cancer, traditional treatment is often ineffective. The search for new treatments has led to the discovery of molecules that act against certain types of cancer, resulting in significantly more efficient and specialized alternatives, with a lower risk of side effects [4]. They are mainly related to secondary metabolites, which are byproducts of the primary metabolism of cells and exhibit multiple uses for industry, agriculture, and medicine [5]. Bacteria and fungi are producers of secondary metabolites, whose expression may be regulated by modifying light, stress, culture conditions, and symbiosis, or exposure to certain pathogens [6].

Plant endophytic fungi are key candidates in the search for bioactive molecules to treat human diseases. A variety of fungal populations can be observed in different plants synergistically living and being exposed to diverse conditions, which possess the potential to produce compounds that may be different from those they normally make. These new compounds have been studied as candidates for the treatment of diseases such as cancer [7,8].

Endophytic fungi of cacti, such as *Lophocereus marginatus*, produce metabolites against diverse types of cancer, particularly lymphoma. They include *Penicillium citrinum*, *Aspergillus versicolor*, *Metarhizium anisopliae*, and *Cladosporium* sp. [9]. This represents an opportunity to discover new secondary metabolites with therapeutic potential. Therefore, this work aimed to evaluate the in vitro and in vivo antitumor activity of crude extracts from the *L. marginatus* endophytes *A. versicolor* and *M. anisopliae* against murine lymphoma. 

## 2. Materials and Methods

### 2.1. Fungi Strains

We used the fungal strains *A. versicolor* PME-H005 and *M. anisopliae* PME-H007, endophytes of *L. marginatus* [9]. They were activated on potato dextrose agar (PDA; Difco Laboratories, Detroit, MI, USA) for 10 d (PME-H007) or 12 d (PME-H005) at 28 °C ± 2 °C. 

### 2.2. Fermentation and Extract Preparation

To evaluate the production of metabolites, we inoculated two-liter flasks containing one liter of culture media with eight 0.8 cm^2^ fragments of fresh mycelium. PME-H005 was cultured on malt broth (13 g/L of maltose, 5.5 g/L of casein peptone, and 0.5 g/L of yeast extract; Sigma-Aldrich, St. Luis, MO, USA) with a pH of 4.7 for 14 d without agitation at 28 °C ± 2 °C and PME-H007 was cultured on potato dextrose broth (PDB; Difco Laboratories) at pH 5.7 ± 0.2, under constant stirring at 28 °C ± 2 °C and 150 rpm for 7 d, containing 10,000 U/mL penicillin and 10 mg/mL streptomycin (Difco Laboratories). 

After fermentation, biomass and supernatant fluids were separated by filtration. The biomass was dried in an oven at 40 °C for 24 h and milled using a Hamilton Beach grinder (model 80335R; Glen Allen, VA, USA). The biomass of each fungus was subjected to extractions with hexane (Sigma-Aldrich). For PME-H007, the obtained biomass was placed in a Soxhlet extractor with 500 mL of hexane for 48 h. For PME-H005, the extract was obtained by maceration, adding 500 mL of hexane to the biomass and constantly stirring at 150 rpm for 48 h. A liquid–liquid extraction was performed on the supernatant with ethyl acetate (CTR Scientific, Monterrey, México) at a ratio of 1:1. Once the extracts were obtained, they were rotoevaporated (Buchi R-3000; Brinkman Instruments, Inc., Westbury, NY, USA), after which dry extracts were reconstituted with dimethyl sulfoxide (DMSO; Sigma-Aldrich) and stored at −20 °C, until use.

### 2.3. Tumor and Normal Cells

We used murine L5178Y-R lymphoma cells (ATCC CRL-1722) and the control human peripheral blood mononuclear cells (PBMC), obtained from 20 mL of blood from a healthy volunteer donor. The blood was then diluted at a 1:9 ratio with phosphate-buffered saline (PBS) and added to a tube containing 15 mL of Ficoll-Paque PLUS (GE Healthcare Life Sciences, Pittsburgh, PA, USA), ensuring that the mixture did not mix. Next, it was centrifuged at 400× *g* for 30 min at 20 °C, after which the layer of lymphocytes was carefully collected and washed with PBS at 100× *g* for 10 min at 20 °C and the supernatant fluid was removed. This was maintained in RPMI 1640 culture medium (Life Technologies, Inc., Grand Island, NY, USA), supplemented with 10% inactivated fetal bovine serum (Life Technologies, Inc.) (the inactivation was performed in a water bath at 56 °C for 30 min), and 1% antibiotic/antimycotic solution (Life Technologies, Inc.). This medium was referred to as the complete RPMI 1640 culture medium. Cells were cultured at 37 °C in an atmosphere of 5% CO_2_ in 95% air.

### 2.4. Effect of Extracts on L5178Y-R Cell Growth

One hundred microliters of L5178Y-R cell suspensions were cultured at a density of 1 × 10^4^ cells/well and PBMC at 1 × 10^5^ cells/well into flat-bottomed 96-well plates (Corning Incorporated, Corning, NY, USA) in complete RPMI 1640 culture medium. After 24 h of incubation, the cells were further incubated in triplicate for 48 h at 37 °C in 5% CO_2_, with 1:2 serial dilutions of 1 mg/mL stock extracts, resulting in concentrations ranging from 15.625 μg/mL to 250 μg/mL, in a final volume of 200 µL. Tumor cell growth was then evaluated by the colorimetric 3-[4,5-dimethylthiazol-2-yl]-2,5-diphenyltetrazoliumbromide (MTT; Affymetrix, Cleveland, OH, USA) reduction assay by adding 15 µL of MTT (0.5 mg/mL final concentration) and incubating at 37 °C for three additional hours. Formazan crystals were dissolved with DMSO, and optical densities (ODs) were measured at 570 nm in a MULTISKAN GO microplate reader (Thermo Fisher Scientific, Waltham, MA, USA). A purple coloration after dissolving formazan crystals indicates the presence of viable cells. This assay is useful for evaluating proliferation and cytotoxicity [10]. The cell growth inhibition percentage was calculated as follows: % Growth inhibition = 100 − ((OD_570_ in extract-treated cells/OD_570_ in untreated cells) (100)), using 0.05 µg/mL vincristine sulfate (VC; Hospira, Warwickshire, UK) as a positive control. Logarithmic scale concentrations were plotted against percent growth inhibition to determine IC_50_ values, which were used to calculate the selectivity index (SI) by dividing the IC_50_ of normal cells by that of tumor cells [11].

### 2.5. DPPH Assay

The antioxidant activity of each extract was evaluated by the 2,2-diphenyl-1-picrylhydrazyl (DPPH) method, as previously reported [12]. DMSO was used as a negative control and ascorbic acid as a standard at concentrations ranging from 10 µg/mL to 100 µg/mL. In a 96-well plate, we incubated 100 μL of extract at different concentrations and 100 μL of DPPH (Sigma-Aldrich) for 30 min at room temperature in darkness. ODs were then read at 517 nm in a MULTISKAN GO microplate reader (Thermo Fisher Scientific) and the percentage of inhibition of the DPPH radical was calculated.

### 2.6. Hemolytic and Anti-Hemolytic Activity

The hemolytic and anti-hemolytic activity of the extracts was determined by a previously reported technique [13]. We obtained 20 mL of blood from a healthy volunteer in tubes with EDTA anticoagulant, after which the red blood cells were washed three times with PBS at pH 7.2 and 5% erythrocytes were suspended in sterile PBS. Next, we incubated the extracts (15.625 µg/mL to 250 µg/mL) plus the erythrocyte suspension in 2 mL tubes in triplicate, using distilled water as a positive control and PBS as a negative control, at 37 °C for 30 min, after which we centrifuged them at 4 °C for 5 min at 13,000 rpm. To evaluate the anti-hemolytic activity, we incubated a red blood cell suspension with 150 mM 2,2′-azobis(2-amidinopropane) dihydrochloride (AAPH; Sigma-Aldrich) plus extracts, using PBS as a negative control and erythrocytes with AAPH as a positive control, at 37 °C for 5 h at 200 rpm and centrifuged them under the conditions described above. In both cases, once the sample was centrifuged, 200 μL of the supernatant was obtained and placed in a 96-well microplate to measure ODs at 540 nm.

The percentage of hemolysis and anti-hemolysis was calculated using the following formulas:

$$\%\;\mathrm{Hemolysis}=\frac{{\mathrm{OD}}_{540}\;\mathrm{treatment}\;-{\mathrm{OD}}_{540}\;\mathrm{negative}\;\mathrm{control}}{{\mathrm{OD}}_{540}\;\mathrm{positive}\;\mathrm{control}\;-{\mathrm{OD}}_{540}\;\mathrm{negative}\;\mathrm{control}}\;\times \;100$$

$$\%\;\mathrm{AAPH}\;\mathrm{inhibition}=\;1-(\frac{{\mathrm{OD}}_{540}\;\mathrm{treatment}\;-{\mathrm{OD}}_{540}\;\mathrm{negative}\;\mathrm{control}}{{\mathrm{OD}}_{540}\;\mathrm{positive}\;\mathrm{control}\;-{\mathrm{OD}}_{540}\;\mathrm{negative}\;\mathrm{control}}\;\times \;100)$$

### 2.7. Phytochemical Analysis

The phytochemical profile of the extracts was analyzed through qualitative tests to detect the presence of different compounds, using each extract dissolved in methanol. For the detection of xanthophylls, 0.2 mL of HCl was added to 0.5 mL of extract; a green or purple coloration indicates a positive result. We used Baeyer’s test to identify unsaturated carbon–carbon bonds, where five drops of 1% KMnO_4_ were added to 10 drops of each extract. The test is positive if there is a color change from purple to reddish-brown, along with a precipitate. Carbohydrates were detected by Molisch’s test. For this, the Molisch reagent was added to 10 drops of extract and concentrated H_2_SO_4_; the presence of a purple ring at the interface is interpreted as positive. The presence of flavonoids was detected by Shinoda’s test, where 10 drops of HCl and a small piece of Mg were added to 20 drops of extracts; the presence of an orange, red, or purple color is indicative of a positive test. Alkaloids were detected by Dragendorff’s test, where five drops of Dragendorff’s reagent were added to five drops of extract; the presence of an orange-brown precipitate indicates a positive test. The phenolic hydroxyl group was determined by FeCl_3_ test, where two drops of reagent were added to three drops of extract; the test is positive if a red, blue, or purple color or precipitate appears. For sterols and triterpenes, the Liebermann–Burchard test was used. Ten 10 drops of the Liebermann–Burchard reagent were added to 20 drops of extract; the presence of green or blue colors is positive for sterols, whereas a purple or pink color is positive for triterpenes. Coumarins were detected by adding 10 drops of 10% NaOH to the extract, resulting in a color change, and by slowly adding a drop of concentrated HCl, a discoloration was observed, indicating a positive test. The Baljet test was used to determine the presence of lactones, where five drops of Baljet reagent were added to five drops of extract; the presence of a red, orange, or violet precipitate is a positive result [14,15].

### 2.8. Animals

We used 6- to 7-week-old female BALB/c mice, provided by the vivarium of the UANL Laboratorio de Inmunología y Virología in Facultad de Ciencias Biológicas at Universidad Autónoma de Nuevo León, México. They were housed in microventilated cages enriched with cardboard tubes for recreational purposes, with access to water and food ad libitum, in a stress-free and pathogen-free environment at a temperature of 22 °C, with cycles of light and darkness of 12 h and a relative humidity of 45%. We followed the established guidelines for the care and welfare of animals in cancer research [16]. A clinical score assessing body weight, hair coat condition, posture, and activity was used to determine the animal endpoints.

### 2.9. Maximum Tolerated Dose and Hepatotoxicity of the Extracts

We performed the maximum tolerated dose test, following the Organization for Economic Co-operation and Development (OECD) recommendations [17]. Extracts were suspended in a vehicle consisting of 90% PEG-300 (Sigma-Aldrich), 5% DMSO, and 5% of 96% ethanol [18]. A single dose of 100 mg/kg or 1000 mg/kg for each extract was intraperitoneally administered to each mouse, in addition to maintaining a group treated with vehicle and the control group. The observation period was 14 d and during this period the weight and clinical score of each mouse were monitored. Animals were euthanized by cervical dislocation if there was a weight loss ≥ 20% or a clinical score ≥ 3. We intraperitoneally administered 25 mg/kg to 40 mg/kg sodium pentobarbital (Aranda Salud Animal, Queretaro, México) as an anesthetic. A cardiac puncture was then performed to extract a blood sample, followed by animal euthanasia. The blood samples were stored in tubes without heparin and centrifuged at 3000 rpm for five minutes to separate serum and perform liver function tests [19].

### 2.10. Antitumor Activity of Extracts

The lymphoma was maintained by intraperitoneally inoculating 0.2 mL of L5178Y-R tumor cells (5 × 10^6^ cells/mouse) in 4- to 6-week-old female BALB/c mice, as previously reported [20]. Then, days after the inoculation, ascites were collected from the peritoneal cavity of mice killed by cervical dislocation. After this, the ascitic suspension was placed in a 50 mL tube containing 10 mL of RPMI-1640 medium. The cell suspensions were washed twice in PBS by centrifuging at 2000 rpm for 10 min and adjusted to 2 × 10^5^ cells/mL in PBS, after which female 6- to 7-week-old BALB/c mice received a subcutaneous administration (upper-right thigh) of 0.2 mL of this lymphoma suspension. After 7 d of tumor inoculation, the L5178Y-R tumor-bearing mice were selected and treated with *A. versicolor* and *M. anisopliae* extracts. 

The controls were extract-untreated tumor-bearing mice (untreated), vehicle (a solution containing 90% PEG-300, 5% DMSO, and 5% of 96% ethanol)-treated tumor-bearing mice (vehicle), and normal untreated (untreated control w/o tumor) mice. Overall survival and tumor volume (length) (width)^2^/2 were then determined. Tumor measurements (length and width) were obtained with a vernier caliper. Survival was daily evaluated for a maximum period of 20 d, and we recorded the day of euthanasia for each individual in each group.

### 2.11. Statistical Analysis

Results were expressed as mean ± SD of three replicate determinations per treatment (in vitro study) and five mice (in vivo study) per experimental group. Statistical analyses were performed using the Graph Pad Prism 8 program (GraphPad Software Inc., San Diego, CA, USA). Survival data were processed using the Kaplan–Meier survival analysis followed by a Log-rank test with *p* < 0.05. A two-way analysis of variance was performed on the normally distributed data for weight variation followed by the Tukey test to determine differences between treatments with *p* < 0.05. A one-way analysis of variance was performed on the normally distributed data for tumor volumes followed by the Dunnett’s test to determine differences between treatments and control with *p* < 0.05. 

## 3. Results

### 3.1. In Vitro Antitumor and Biological Activity of A. versicolor and M. anisopliae Extracts Against the Murine Lymphoma L5178Y-R

The yields obtained from the *A. versicolor* and *M. anisopliae* hexane and ethyl acetate extracts are shown in Table 1. *A. versicolor* and *M. anisopliae* hexane and ethyl acetate extracts presented the highest antitumor activity. We observed an IC_50_ < 14 µg/mL for the ethyl acetate extracts of both fungi, as compared with hexane extracts, which were <71 µg/mL (Table 1). *M. anisopliae* possessed the lowest IC_50_ values for both extracts. The ethyl acetate extracts of both fungi showed SIs > 30, whereas hexane extracts had SIs < 4 (Table 1). The positive control with vincristine resulted in 80% lymphoma cell growth inhibition. 

On the other hand, ethyl acetate extracts showed higher antioxidant activity against the DPPH radical, as compared with that of hexane extracts with IC_50_ values < 140.2 µg/mL. The positive control of ascorbic acid showed an IC_50_ value of 7.23 ± 1.35 µg/mL. Regarding hemolytic activity, the hexane extracts of both fungi caused hemolysis of erythrocytes, whereas the ethyl acetate extracts showed hemoprotective effects on erythrocytes with IC_50_ values < 27 µg/mL. 

### 3.2. Phytochemical Analysis of A. versicolor and M. anisopliae Extracts

The phytochemical profile was determined by qualitative tests of each extract (Table 2). The ethyl acetate extract of *A. versicolor* was positive for unsaturated carbon–carbon bond, flavonoids, the phenolic hydroxyl group, and lactones, whereas the hexane extract was positive for unsaturated carbon–carbon bonds, carbohydrates, flavonoids, and the phenolic hydroxyl group. On the other hand, the ethyl acetate extract of *M. anisopliae* contains unsaturated carbon–carbon bonds, carbohydrates, the phenolic hydroxyl group, and lactones, whereas the hexane extract was only positive for unsaturated carbon–carbon bonds and carbohydrates.

### 3.3. Maximum Tolerated Dose of A. versicolor and M. anisopliae Extracts in a Murine Model

Based on the results of the in vitro assays mentioned above, we selected the *A. versicolor* and *M. anisopliae* ethyl acetate extracts for the in vivo study since they have a lower IC_50_ in the L5178Y-R cell line, a higher SI, and possess hemoprotective activity, as compared with hexane extracts.

**Figure 1:**
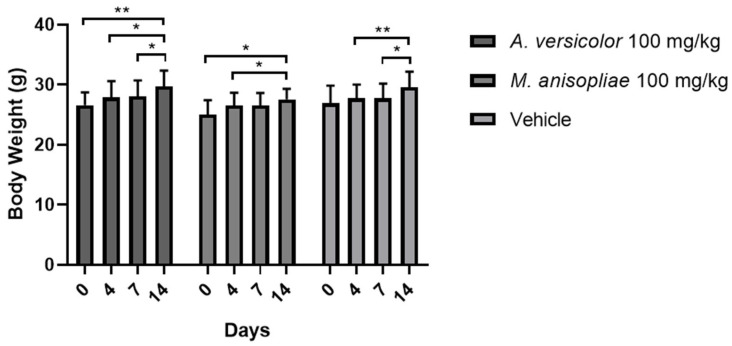
Weight variation in mice treated with extract and vehicle for 14 d. * *p* < 0.05 and ** *p* < 0.01.

To determine the appropriate concentration to administer to mice, the maximum tolerated dose test was performed, using the extracts at 100 and 1000 mg/kg of mouse weight, dissolved in the vehicle. At the 1000 mg/kg dose, mice did not survive for more than one hour after administration. In addition, mice treated with the vehicle and the extracts at 100 mg/kg showed 100% survival at 14 d. We did not observe weight loss in mice treated with the extract and vehicle (Figure 1). The liver function tests performed on each group did not reveal values outside the normal range reported by others, but they were similar to those obtained in the control group (Table 3). Therefore, the concentration of 100 mg/kg was selected as the maximum dose to be administered to mice.

### 3.4. In Vivo Effect of A. versicolor and M. anisopliae Extracts Against Murine Lymphoma L5178Y-R Cells

**Figure 2:**
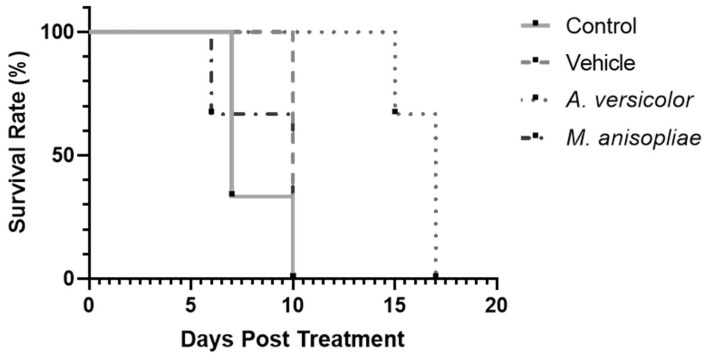
Kaplan–Meier survival curves of female BALB/c mice treated with *A. versicolor* fungi and *M. anisopliae* ethyl acetate extracts, vehicle, and untreated control. Post hoc analysis with the log-rank test showed a significant difference between control and *A. versicolor* treatment survival (*p* = 0.0224).

**Figure 3:**
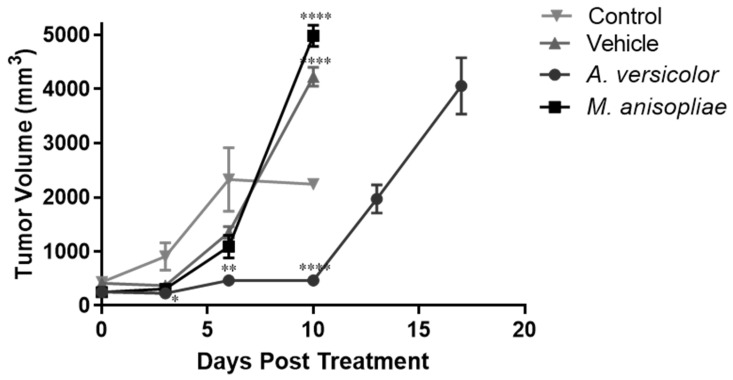
Tumor volumes of female BALB/c mice treated with *A. versicolor* and *M. anisopliae* ethyl acetate extracts, vehicle, and untreated control. * *p* < 0.05, ** *p* < 0.01, and **** *p* < 0.0001, compared with the untreated control using the Dunnett´s test.

After 7 d of tumor inoculation, we intraperitoneally administered three doses of 30 mg/kg per mouse, every two days. Mice in the control group, treated with vehicle and *M. anisopliae* extract, showed a maximum survival of 10 d (Figure 2), presenting a tumor volume > 4000 mm^3^ (Figure 3). In addition, mice treated with *A. versicolor* extract had significantly (*p* = 0.0224) higher survival time (up to 17 d), as compared with the other treatments (Figure 2). Figure 3 shows that the tumor volume of this group did not present a significant increase on day 10, as compared with the other treatments. However, we observed significant (*p* < 0.0001) differences in tumor volumes between treatments on day 10 (Figure 3).

## 4. Discussion

The heterogeneity in the clinical outcome of cancer patient treatment and the characteristics of distinct types of cancer have caused the morbidity and mortality rate of this disease to increase in recent years [23]. Based on this issue and the co-evolution of treatments and cancer, numerous studies have focused on the search for novel technologies, molecules, and strategies for the treatment of cancer, either directly attacking it or optimizing existing strategies to improve their effectiveness [24].

Fungal extracts represent an important alternative to finding or reinforcing possible treatments for several types of cancer since it has been proven that many species possess anticancer properties through different mechanisms [25]. We evaluated the in vitro and in vivo antitumor activities of hexane and ethyl acetate extracts from the endophytic fungi *A. versicolor* and *M. anisopliae*, which presented IC_50_ values lower than those reported in a previous study, in which the same strains were used but with methanol extracts, where the IC_50_ values were 166.2 ± 1.8 µg/mL for *A. versicolor* and 132.9 ± 1.5 µg/mL for *M. anisopliae* [26]. In the present study, we showed IC_50_ values ranging from 13 µg/mL to 71 µg/mL for *A. versicolor* and 9 µg/mL to 45 µg/mL for *M. anisopliae*. The ethyl acetate extracts of both fungi showed a lower IC_50_ in the tumor cells, in addition to a higher SI, as compared with the hexane extracts, these being > 30 for the ethyl acetate extracts and around 3, for the hexane extracts. This difference between each type of extract may be due to the polarity characteristics of the solvents used and the affinity of the compounds produced by the fungi towards these solvents. This allows for the separation of different compounds according to their polarity, which directly impacts biological activity and therefore higher lymphoma cell growth inhibition and significantly lower harmful effects on healthy cells [27].

On the other hand, the IC_50_ reported for *A. versicolor* ethyl acetate extract was 13.51 ± 1.62 µg/mL for the L5178Y-R cell line; comparable with that reported by Sajna et al. [28] using *Aspergillus* ethyl acetate extracts against HeLa and MCF7 cells, with IC_50_ values of 13.46 and 18.75 µg/mL, respectively. Other authors have also demonstrated the cytotoxic activity of other strains of endophytic *A. versicolor* and *M. anisopliae* against diverse types of cancer [29,30].

The *Aspergillus* genus is an important producer of metabolites with different bioactivities, including antitumor effects, as in colorectal cancers [31]. At least 330 compounds with antiviral, antimicrobial, and cytotoxic activities produced by *A. versicolor* have been reported, mainly belonging to the xanthone, anthraquinone, peptide, diketopiperazine, terpene, and alkaloid families of molecules, highlighting alkaloids and diketopiperazines as the most abundant of them (around 42.6%) [32].

*M. anisopliae* has also been shown to affect breast cancer cells mediated by anisoplin. In a previous report, recombinant anisoplin was shown to be cytotoxic against MCF-7 cells by apoptosis [33].

*A. versicolor* and *M. anisopliae* antitumor activities have been previously reported by others. Our results demonstrated the pharmacological potential of their extracts since crude extracts with IC_50_ values < 100 µg/mL may be purified to obtain compounds with higher activity [34].

Cells produce free radicals as part of their metabolism, which may cause oxidative damage and increase the risk of suffering from diseases such as cardiovascular disorders, diabetes, and cancer. Antioxidants are compounds that prevent oxidative damage to the cell [35]. We observed that *A. versicolor* ethyl acetate extract has antioxidant activity against the DPPH radical (Table 1), which has been previously reported [36]. The antioxidant activity of ethyl acetate extracts has also been reported for other endophyte species of the genus *Aspergillus*, such as kojic acid-producing *A. flavus* with an IC_50_ value of 99.3 μg/mL [37]. The *M. anisopliae* ethyl acetate extract also has antioxidant activity, which agrees with a previous report by Shin et al. [38], showing that 23 isolates of *M. anisopliae* possess antioxidant activity. The presence of compounds with antioxidant activity may synergistically act with other chemotherapeutic or radioactive treatments, generating a protective effect on healthy cells, improving antitumor activity, and indirectly reducing side effects [39].

The evaluation of hemolysis is important to determine the cytotoxicity of a compound or extract that will be evaluated in a biological system [40]. It was observed that the ethyl acetate extracts of both fungi possessed anti-hemolytic activity with IC_50_ values < 27 µg/mL, whereas the hexane extracts produced hemolysis of erythrocytes. It has been reported that *A. versicolor* of marine origin produces an enzyme called versiase that does not have hemolytic effects and has anticoagulant and thrombolytic activity [41]. It was also shown that metacytophilin from *Metarhizium*, does not have hemolytic effects [42].

Our results suggest that ethyl acetate extracts have compounds that reduce the oxidative damage caused by AAPH, which prevents the rupture of the erythrocyte membrane [43]. Furthermore, these extracts have higher antioxidant potential against the DPPH radical, as compared with hexane extracts, which may indicate the presence of secondary metabolites with strong antioxidant activity. Because most chemotherapy treatments are intravenously administered, it is crucial to discard hemolytic effects. Therefore, compounds with antioxidant activity are important to protect erythrocytes and other cells in the bloodstream, thus preventing anemia [44].

As mentioned above, the polarity of each solvent may attract different metabolites. The heterogeneity in the compounds produced by every strain of fungus is variable due to the nature and culture conditions, which facilitates the isolation of new compounds. Until 2020, at least 330 different compounds produced by *A. versicolor* have been described, the main ones being peptides and diketopiperazines, followed by alkaloids, terpenes, anthraquinones, polyketides, lactones, and diphenyl ethers [32,45]. According to our phytochemical profile of extracts of endophytic *A. versicolor* from *L. marginatus*, we found compounds of flavonoid and phenolic nature, as well as lactones in the case of the ethyl acetate extract and carbohydrates in the hexane extract. The presence of lactones produced by *A. versicolor* has been reported in extracts with ethyl acetate, which agrees with our profile [30].

On the other hand, *M. anisopliae* has been reported as a producer of compounds such as peptides, polyketides, terpenoids, carboxylic acids, and alcohols [46]. Specifically, ethyl acetate extracts from *M. anisopliae* have been reported to contain phenols, amines, and carboxylic acids [47]. Our ethyl acetate extract from *M. anisopliae* contains metabolites of phenolic nature, carbohydrates, unsaturated carbon–carbon bonds, and lactones. In contrast, our hexane extract possesses carbohydrates and unsaturated carbon–carbon bonds, suggesting that the presence of compounds such as phenols and lactones may be responsible for the antitumor activity of the ethyl acetate extracts of *M. anisopliae*.

In our in vivo experiments, it was observed that *A. versicolor* ethyl acetate extract did not allow tumor volume growth during the first 10 d and increased survival, as compared with the untreated control group (Figure 2 and Figure 3), extending it to 17 d, which represents a longer survival time as reported with the L5178Y-R cell line using vincristine, with a maximum survival of 15 d [48]. On the other hand, in the case of *M. anisopliae* ethyl acetate extracts and the vehicle group, we did not observe an increase in survival, as compared with the untreated control (Figure 2). In vivo studies in L5178Y-R lymphoma models have shown that crude extracts of *Lophocereus schottii* reduce tumor volume and increase survival in BALB/c mice [49].

The in vivo efficacy of extracts from different fungi against several types of cancer reveals promising results as an alternative treatment since they represent important sources of compounds with anticancer properties [50].

## 5. Conclusions

The *A. versicolor* and *M. anisopliae* hexane and ethyl acetate extracts inhibited L5178Y-R murine lymphoma cell growth in vitro. The ethyl acetate extracts were also shown to possess antioxidant and hemoprotective activities. Furthermore, the *A. versicolor* ethyl acetate extract showed a significant increase in tumor-bearing mice survival; additional studies are needed to determine the bioactive compounds responsible for the anticancer effect.

## Figures and Tables

**Table 1:** The yield and in vitro biological activity of *A. versicolor* and *M. anisopliae* extracts.

	Yield	L5178Y-R Cell Growth Inhibition ^a^	PBMC Growth Inhibition ^a^	Selectivity Index	Antioxidant Activity ^a^	Hemolysis ^a^	Anti-Hemolysis ^a^
*A. versicolor*							
Ethyl acetate	29.3 mg/L ^b^	13.51 ± 1.62	467.4 ± 0.69	34.59	140.2 ± 1.4	>800	11.5 ± 1.6
Hexane	42.81 mg/g	70.52 ± 1.6	180.8 ± 1.57	2.56	>250	83.28 ± 1.9	>250
*M. anisopliae*							
Ethyl acetate	100.1 mg/L	9.168 ± 1.21	348.0 ± 1.23	37.95	111.7 ± 1.5	>800	26.4 ± 1.6
Hexane	21.11 mg/g	44.93 ± 1.58	146.9 ± 1.59	3.26	>250	84.89 ± 1.4	>250

^a^ IC_50_ (μg/mL). ^b^ Data represent the means ± SD; vincristine resulted in 80% lymphoma cell growth inhibition.

**Table 2:** Phytochemical analysis of *A. versicolor* and *M. anisopliae* extracts.

Assay	*A. versicolor*		*M. anisopliae*	
	Ethyl Acetate	Hexane	Ethyl Acetate	Hexane
Xanthophyll	Negative	Negative	Negative	Negative
Baeyer	Positive	Positive	Positive	Positive
Molisch	Negative	Positive	Positive	Positive
Shinoda (flavonoids)	Positive	Positive	Negative	Negative
Dragendorff (alkaloids)	Negative	Negative	Negative	Negative
FeCl_3_ (phenolic hydroxyl group)	Positive	Positive	Positive	Negative
Liebermann–Burchard	Negative	Negative	Negative	Negative
Coumarin	Negative	Negative	Negative	Negative
Baljet	Positive	Negative	Positive	Negative

**Table 3:** Liver function tests on mice treated with *A. versicolor* and *M. anisopliae* extracts, vehicle, and untreated control.

	*A. versicolor* (100 mg/kg)	*M. anisopliae* (100 mg/kg)	Vehicle	Untreated Control	Reference Value
Albumin *	3.1	3.3	3.3	3.4	3.1–5.3 ^a^
Total proteins *	5.1	5.6	5.3	5.5	5.23 ± 1.68 ^b^
Alkaline phosphatase **	361	428	365	399	362.9 ± 226.6 ^b^
Aspartate aminotransferase **	137	341	277	122	67–381 ^a^
Alanine transaminase **	49	145	182	60	40–170 ^a^

Units were expressed in g/dL (*) and U/L (**). ^a^ [21] and ^b^ [22].

## Data Availability

The datasets generated and/or analyzed during the present study are available from the corresponding author on reasonable request.
